# Digitoxin Affects Metabolism, ROS Production and Proliferation in Pancreatic Cancer Cells Differently Depending on the Cell Phenotype

**DOI:** 10.3390/ijms23158237

**Published:** 2022-07-26

**Authors:** Heléne Lindholm, Katarina Ejeskär, Ferenc Szekeres

**Affiliations:** Biomedicine, School of Health Sciences, University of Skövde, 54145 Skövde, Sweden; helene.lindholm@his.se (H.L.); katarina.ejeskar@his.se (K.E.)

**Keywords:** pancreatic cancer, digitoxin, cardiac glycosides, PDAC, KRAS, metabolism, ROS, cell proliferation

## Abstract

Digitoxin has repeatedly shown to have negative effects on cancer cell viability; however, the actual mechanism is still unknown. In this study, we investigated the effects of digitoxin (1–100 nM) in four pancreatic cancer cell lines, BxPC-3, CFPAC-1, Panc-1, and AsPC-1. The cell lines differ in their KRAS/BRAF mutational status and primary tumor or metastasis origin. We could detect differences in the basal rates of cell proliferation, glycolysis, and ROS production, giving the cell lines different phenotypes. Digitoxin treatment induced apoptosis in all four cell lines, but to different degrees. Cells derived from primary tumors (Panc-1 and BxPC-3) were highly proliferating with a high proportion of cells in the S/G2 phase, and were more sensitive to digitoxin treatment than the cell lines derived from metastases (CFPAC-1 and AsPC-1), with a high proportion of cells in G0/G1. In addition, the effects of digitoxin on the rate of glycolysis, ROS production, and proliferation were dependent on the basal metabolism and origin of the cells. The KRAS downstream signaling pathways were not altered by digitoxin treatment, thus the effects exerted by digitoxin were probably disconnected from these signaling pathways. We conclude that digitoxin is a promising treatment in highly proliferating pancreatic tumors.

## 1. Introduction

Pancreatic ductal adenocarcinoma (PDAC) is a tumor in the exocrine part of the pancreas [1]. It has one of the poorest prognoses of all cancer forms, with a five-year survival rate of less than 5% [2]. The currently used therapies have considerable adverse side effects on unspecific targets, including healthy normal cells [3]. Hence, new treatment regimens specifically affecting cancer cells are urgently needed. There are differences between normal cells and cancer cells that are distinct and common for all cancers, such as mutations in oncogenes and tumor suppressor genes, metabolic alterations, aberrant production of reactive oxygen species (ROS), and enhanced proliferation [4,5]. Normal cells have a strictly regulated proliferation controlled by mitogenic signaling [6]. Cancer cells with mutations in oncogenes and tumor suppressor genes proliferate at a high rate, without any mitogenic signaling [7]. Cancer cells also have an increased rate of metabolism with subsequent high ROS production, which affects the proliferation rate [8].

Digitoxin is a well-known cardiac glycoside [9,10,11] with the potential to act as a potent anti-cancer drug in the therapeutic concentration range of 25–40 nM [10,12,13]. Epidemiological data suggest that patients who received treatment with the cardiac glycosides digoxin or digitoxin have a higher survival rate and lower recurrence in different cancer malignancies [14,15]. Cancer cells seem to be more sensitive to digitoxin compared with normal cells, but the reason for this is still unknown [14]. Cardiac glycosides inhibit the Na^+^/K^+^-ATPase by binding to the subunit α, blocking its function. Increased intracellular levels of sodium activates the Na^+^/Ca^2+^-exchanger to pump sodium out of the cell and calcium into the cell [16]. The increase in intracellular calcium has several effects on cells. The impaired Ca^2+^ retention affects energy metabolism in the mitochondria, causing an increase in oxidative phosphorylation and in the production of ROS [17]. Intracellular redox status is indispensable for normal activities in cells and is affected by the accumulation of ROS [18]. The redox status is important for the cell signaling pathways, both in survival and apoptosis, but also for regulation and progression of the cell cycle [19,20,21,22,23,24].

Assessment of the proliferation rate gives information about the aggressiveness, and the prognosis of the cancer is an important step in the evaluation of the effects of different drugs and treatments [25]. The cell cycle is divided into four phases, Gap 1 (G1), synthesizing (S), Gap 2 (G), and mitosis (M). During the cell cycle, the signaling dependence on redox status changes and the effects of ROS on cell proliferation vary [26]. Cells in the resting, non-proliferating phase G0 have a basal oxidative metabolism. Migrating and invading cells are mostly found in this phase [27]. Proliferating cells leave the G0 phase and enter the G1 phase, where the metabolism shifts to glycolysis in order to meet the demands of RNA and protein synthesis [28]. Cells in the S/G2 phase turn to glutaminolysis and mitochondrial oxidative phosphorylation for their ATP production and nucleic acid synthesis. The high rate of oxidative phosphorylation is connected with the subsequent increase in the production of ROS [29]. ROS production increases during the cell cycle, with the highest production in the G2/M phase [26]. Cancer cells produce more ROS and tolerate higher concentrations than normal cells. When ROS reach too high levels, even cancer cells are affected and cell division is stalled. After a prolonged arrest, cells die from apoptosis [26,30]. Drugs and stressors in the surrounding area can affect cells through an increased production of ROS, and thereby cause an imbalance in redox status and oxidative stress, leading to cell cycle arrest [22,31].

Beyond its role as an ion channel, Na^+^/K^+^-ATPase is involved in intracellular signaling pathways. According to Tian et al., 2006, Na^+^/K^+^-ATPase functions as a key member of the signaling complex together with the non-receptor tyrosine kinase (Src kinase), which is inactive when complex bound [32,33,34]. When cardiac glycosides bind to Na^+^/K^+^-ATPase, Src kinase dissociates from the complex and becomes activated, resulting in phosphorylation of the epidermal growth factor (EGFR) and activation of the downstream genes such as KRAS, and further the signaling pathways Raf/MEK/ERK and PI3K/Akt [34,35]. Oncogenic signaling is intertwined with metabolic pathways, redox balance, and cell proliferation [7,28]. *KRAS* is the most frequently mutated oncogene in pancreatic cancer, and is mutated in more than 90% of all cases [36]. *BRAF* is another gene mutated in PDAC. Because of its action in the same signaling pathway, a mutated *BRAF* gene never occurs together with a mutation in *KRAS* [37,38]. Mutations in *KRAS* or *BRAF* gives fast proliferating cells a survival advantage in environments with a low supply of oxygen and nutrients, with an increase in glycolytic activity [39,40]. Of the mutations in *KRAS,* 98% occur at position G12, with the most common variants being *KRAS ^p.G12D^* and *KRAS ^p.G12V^* [40]. *KRAS* interacts with and activates the signaling pathways of PI3K/Akt and RAF/MEK/ERK [41]. Through these pathways, signals from the cell surface are transduced to effectors inside the cell, affecting metabolism, apoptosis, and proliferation [42]. Mutated *KRAS* affects many genes important for glycolysis. Hexokinase 2 (HK2) and pyruvate kinase (*PK*), which mediate the first and the last step in glycolysis, respectively, are often up-regulated in cancer cells. The M2 isoform of PK (*PKM2*) has been associated with tissue repair and fast proliferating cells [43]. Mutations in *KRAS* up-regulate the expression of the glucose transporter 1 (GLUT1)*,* which leads to its constitutive activation and an increased uptake of glucose [44,45,46]. Cancer cells with a high rate of aerobic glycolysis have an excess production of lactate [47]. Lactate dehydrogenase A (LDHA) converts pyruvate to lactate and this enzyme is crucial for a high glycolytic metabolism in the absence of oxygen [45]. LDHA is often up-regulated in invasive cancer cells with a high glycolytic rate and is important for cell proliferation in cancer [48].

Here, we used four well-characterized pancreatic cancer cell lines with a different mutational status and origin (primary tumor or metastasis) to cover as many variations as possible in PDAC, so as to study the effects of digitoxin. There were major differences in the basal phenotypes, metabolism, ROS production, and proliferation pattern in the four cell lines. The basal proliferative phenotype was important for the response to digitoxin treatment. Highly proliferating cancer cells were more vulnerable to the toxic effects of digitoxin. The effects seemed to be exerted via the increase in calcium and the resulting effects on the ROS concentration and cell proliferation. In our study, the signaling pathways PI3K/Akt and Raf/MEK/ERK1/2 were not affected by digitoxin treatment.

## 2. Results

### 2.1. Digitoxin Treatment Decreased Pancreatic Cancer Cell Viability and Induced Apoptosis

**Figure 1:**
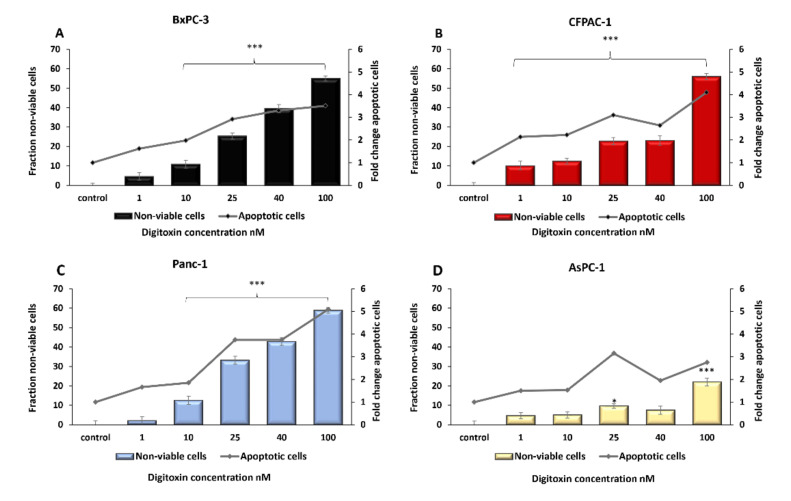
PDAC fraction of non-viable cells (1-MTS) and level of apoptosis (Caspase 3/7) after digitoxin treatment. Cell lines (**A**) BxPC-3, (**B**) CFPAC-1, (**C**) Panc-1, and (**D**) AsPC-1 incubated with digitoxin at concentrations of 1–100 nM for 48 h, with the controls only incubated with complete cell media. The number of viable cells was measured using the MTS assay. Apoptosis was measured using the Caspase 3/7 assay. The fraction of non-viable cells and apoptotic cells for each treatment was normalized to untreated cells (control). Error bars = SEM, Student’s *t*-test * *p* ≤ 0.05, and *** *p* ≤ 0.001.

Treatment with digitoxin at concentrations between 10–100 nM during 48 h incubation had negative effects on cell viability in all four cell lines (Figure 1A−D). The sub-therapeutic concentration of 10 nM digitoxin resulted in a decrease in the number of viable cells compared with control cells, ranging between 12.6% (±2.1, *p* < 0.001) in Panc-1 (Figure 1C) to 11.9% (±2.1, *p* < 0.001) in BxPC-3 (Figure 1A) compared with the respective controls. In the therapeutic range (25–40 nM), the number of viable cells was more decreased, which resulted in between 42.8% (±2.0, *p* < 0.001) in Panc-1, 40 nM (Figure 1C) to 22.8% (±1.8, *p* < 0.001) in CFPAC-1 for 25–40 nM (Figure 1B). The digitoxin induced a reduction in the number of viable cells, with 9.7% in the AsPC-1 treated with 25 nM digitoxin compared with the control (±1.2, *p* = 0.01) and 22.0% in the samples treated with the highest concentration of digitoxin (100 nM) (±1.9, *p* < 0.001). In parallel with these results, there was an increase in apoptotic cells in the samples treated with digitoxin (Figure 1A−D). For concentrations of digitoxin within the therapeutic range (25–40 nM), there was an increase of caspase 3/7, 2.9-fold in BxPC-3 for 25 nM (±0.62, *p* = 0.005), 2.6-fold in CFPAC-1 for 40 nM (±0.65, *p =* 0.0497) and 3.7-fold in Panc-1 for 25 nM (±0.31, *p* = 0.002), compared with the untreated control cells (Figure 1A−C). No significant change in apoptosis was seen in any of the digitoxin concentrations in the AsPC-1 cell line (Supplementary material, Table S1).

### 2.2. Digitoxin Affects the Rate of Aerobic Glycolysis and Increase Intracellular ROS

**Figure 2:**
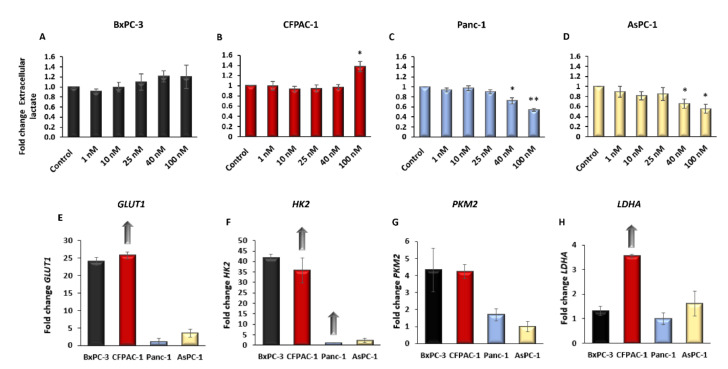
Extracellular lactate as a measure of aerobic glycolysis in PDAC cells after 48 h of digitoxin treatment and the basal expression of genes involved in glycolysis. Extracellular lactate produced by glycolysis measured in the media after 48 h of treatment with digitoxin, normalized to the untreated cells (control) in (**A**) BxPC-3, (**B**) CFPAC-1, (**C**) Panc-1, and (**D**) AsPC-1. The relative basal transcriptional expression (control) of (**E**) *GLUT1*, (**F**) *HK2*, (**G**) *PKM2*, and (**H**) *LDHA*. A regression analysis was done for the transcriptional expression after digitoxin treatment, a significant increase in gene expression with digitoxin treatment is denoted with an arrow. The relative basal transcriptional expression level (delta Ct value, reference gene *PMM1*) is shown as fold change compared with the cell line with the lowest expression. Arrow denotes a significantly increased expression with digitoxin treatment. Error bars = SEM, Student’s *t*-test * *p* ≤ 0.05, ** *p* < 0.01.

Digitoxin treatment affected the rate of glycolysis differently in the four pancreatic cancer cell lines. For BxPC-3, no significant change in glycolysis could be observed with digitoxin treatment when analyzing the extracellular lactate levels (Figure 2A). CFPAC-1 cells treated with 100 nM digitoxin showed an altered glycolytic rate, with a 37% increased extracellular concentration in lactate in the cells treated with 100 nM digitoxin compared with the untreated cells (±0.09, *p* = 0.019); however, in the therapeutic range (25–40 nM), no change was seen (Figure 2B). Panc-1 and AsPC-1 showed a decreasing concentration of extracellular lactate with 40−100 nM digitoxin treatment (±0.09, *p* = 0.003). The rate of glycolysis in Panc-1 was reduced to 54% (±0.03, *p* < 0.001) and in AsPC-1 to 55% (±0.09, *p =* 0.003) when the cells were treated with 100 nM digitoxin, and with 40 nM digitoxin treatment, the rate of glycolysis was reduced to 72% (±0.06, *p =* 0.01 (Panc-1)) and 66% (±0.09, *p* = 0.009 (AsPC-1)) compared with the untreated cells (Figure 2C,D). A clear distinction between the cell lines could be seen in the basal rate of glycolysis, as follows: BxPC-3 (17 mM), CFPAC-1 (25 mM), Panc-1 (12 mM), and AsPC-1 (9 mM). 

Concerning the genes critical for glycolysis, *GLUT1*, *HK2*, and *PKM2*, a higher basal transcriptional expression was seen in BxPC-3 and CFPAC-1 compared with Panc-1 and AsPC-1, which supports the results from the lactate assay (Figure 2E–G). LDHA, catalyzing the last step in aerobic glycolysis, was expressed the highest in CFPAC-1 compared with the other three cell lines (Figure 2H). In CFPAC-1, an increased gene expression was found for all of the genes tested, except for *PKM2*, with higher digitoxin concentrations. No change in expression could be seen with digitoxin treatment in AsPC-1 and BxPC-3 (Figure 2E–H). Panc-1 had an increased expression of *HK2* with increasing concentrations of digitoxin (Figure 2F) (Supplementary Figure S1).

**Figure 3:**
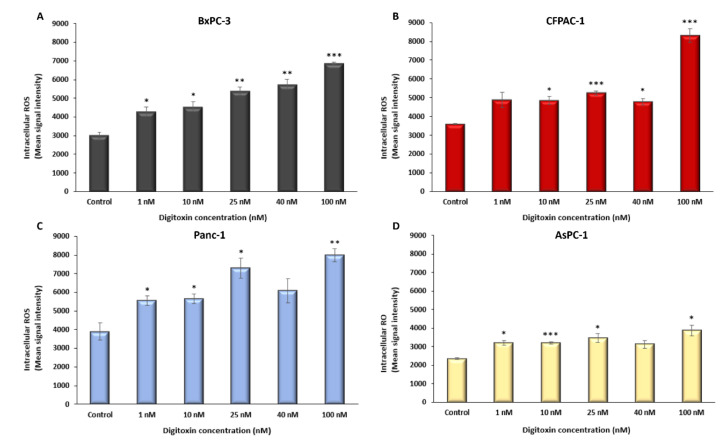
Intracellular ROS in PDAC cells after 48 h of digitoxin treatment and for the controls without digitoxin. (**A**) BxPC-3, (**B**) CFPAC-1, (**C**) Panc-1, and (**D**) AsPC-1 intracellular ROS as the mean signaling intensity. Digitoxin concentrations in nM on the x axis. ROS values normalized to MTS and the control for each cell line. Error bars = SEM, Student’s *t*-test * *p* ≤ 0.05, ** *p* ≤ 0.01 and *** *p* ≤ 0.001.

Intracellular ROS concentrations increased significantly with digitoxin treatment in all four cell lines (Figure 3A–D). In BxPC-3 and Panc-1, the increase in intracellular ROS was 1.8 and 1.9, respectively, in the therapeutic concentration for 25 nM (±0.07, *p* = 0.002 (BxPC-3), ±0.14, *p* = 0.015 (Panc-1)), and 1.5-fold in CFPAC-1 and AsPC-1 (±0.03, *p* < 0.001 (CFPAC-1), ±0.10, *p* = 0.037 (AsPC-1)) for the same digitoxin concentration. At the supratherapeutic concentration of 100 nM, intracellular ROS increased 2.3-fold in BxPC-3 and CFPAC-1, 2.0 in Panc-1, and 1.6 in AsPC-1 (±0.02, *p* = 0.02 (BxPC-3), ±0.10, *p* < 0.001 (CFPAC-1), ±0.09, *p* = 0.002 (Panc-1), ±0.11, *p* = 0.02 (AsPC-1)). 

### 2.3. The Cell Cycle Is Affected by Digitoxin Treatment

**Figure 4:**
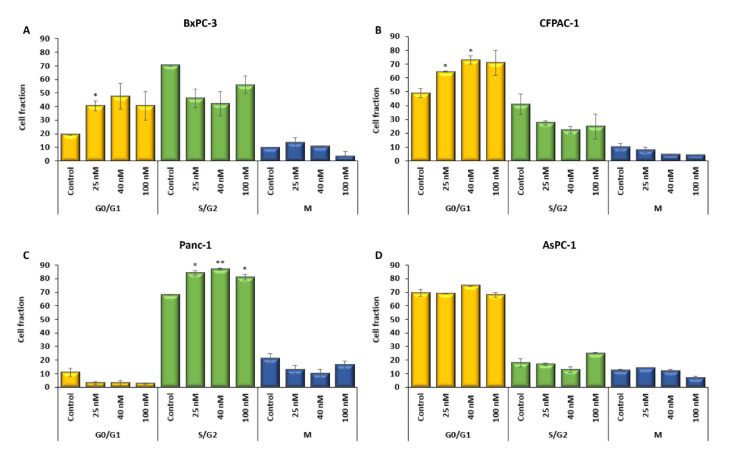
Cell proliferation in PDAC cells in vitro assessed with the Cell-Clock assay (Bio-Color, UK). (**A**) BxPC-3, (**B**) CFPAC-1, (**C**) PAnc-1, and (**D**) AspC-1. Proportions of cells in different phases—for the control and with digitoxin treatment (25 nM, 40 nM, and 100 nM) for 48 h. G0/G1 (yellow), S/G2 (green), and M (blue). Error bars = SEM, Student’s *t*-test * *p* ≤ 0.05; and ** *p* ≤ 0.01.

The proportion of cells in the different stages of the cell cycle differed in the basal conditions between the four cell lines. A high proportion of cells were in the S/G2 phase in BxPC-3 (70.5%) and Panc-1 (68%) compared with CFPAC-1 (41%) and AsPC-1 (18%) (Figure 4). Only 11% of Panc-1 cells were in G0/G1 (Figure 4C); this was low compared with the other three cell lines, BxPC-3 (19.5%), CFPAC-1 (49%), and AsPC-1 (69.5%) (Figure 4A–B,D). 

Following digitoxin treatment for 48 h, both BxPC-3 and CFPAC-1 showed a significant increase in the proportion of cells in G0/G1 at 40.5% (±3.5, *p =* 0.027, 25 nM BxPC-3), 64.5%, and 73.0% (±0.5, *p* = 0.046, 25 nM CFPAC-1 and ±3.0, *p* = 0.034, 40 nM CFPAC-1) (Figure 4A,B). In Panc-1, the proportion of cells in the S/G2 phase increased to 84.0%, 87.0%, and 81.0% with digitoxin treatment (±2.0, *p* = 0.015, 25 nM, ±0.5, *p* < 0.001, 40 nM, ±2.0, *p =* 0.023, 100 nM) (Figure 4C). AsPC-1 had a very low proportion of cells in the S/G2 phase, only 18% in the basal conditions, and showed no significant changes with digitoxin treatment (Figure 4D) (Supplementary Table S2).

### 2.4. Oncogenic Signaling in the PDAC Cell Lines

**Figure 5:**
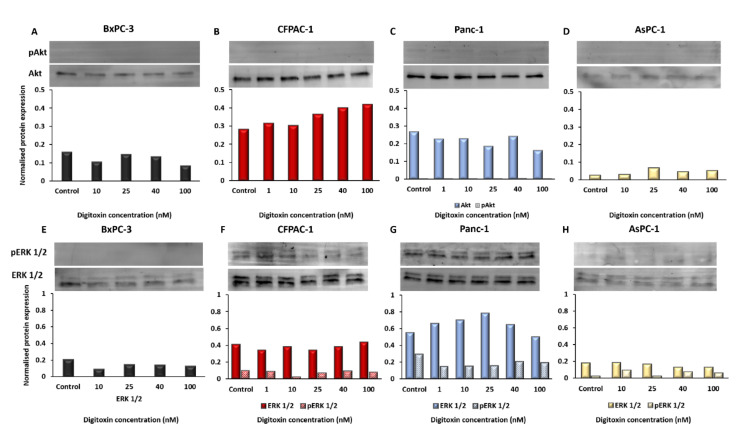
Protein expression of Akt, pAkt, ERK1/2, and pERK1/2 normalized to the total protein for each sample. Untreated cells (control) compared to cells treated with digitoxin for 1−100 nM for 48 h. In (**A**,**E**) BxPC-3, (**B**,**F**) CFPAC-1, (**C**,**G**) Panc-1, and (**D**,**H**) AsPC-1.

In order to analyze the basal PI3K/Akt and/or Raf/MEK/ERK activity in the cell lines and the possible effects of digitoxin treatment, we evaluated the protein levels of pAkt, Akt, pErk1/2, and Erk1/2 after 48 h of treatment and controls (Figure 5A–H). The Western blot analysis showed no significant change in the protein expression after digitoxin treatment, except for the total Akt levels in CFPAC-1, and there was a significant increase in the Akt-level with increasing the concentrations of digitoxin (F1,4, *p* = 0.002) (Figure 5B). Only Panc-1 had detectable levels of phosphorylated Akt (S473) (pAkt), but the protein level was very low and was not affected by digitoxin treatment (Figure 5C).

ERK1/2 was expressed in all four cell lines, and the active form, pERK1/2, was detectable in CFPAC-1, Panc-1, and AsPC-1 (Figure 5F–H); however, the pERK1/2 and ERK1/2 levels were not significantly affected by digitoxin treatment in any of the cell lines. The total level of both Akt and ERK1/2 was higher in CFPAC-1 and Panc-1 compared with the other cell lines (Supplementary Figure S2).

## 3. Discussion

We have focused on the potential use of an already established cardiotonic drug, digitoxin, for the purpose of treating pancreatic cancer. Digitoxin has fatal effects on cell viability and it induced apoptosis to different degrees in the four pancreatic cancer cell lines examined in this study. These results are in accordance with previous studies using cardiac glycosides in human glioma and pancreatic cancer cell lines [49,50]. In an earlier study, we found intracellular calcium was significantly increased in the cell lines BxPC-3 (120%), CFPAC-1 (117%), and Panc-1 (160%) treated with digitoxin at therapeutic concentrations of 25–40 nM. In AsPC-1 cells, only the supra-therapeutic concentration, 100 nM, induced a significant increase in intracellular calcium with 12% (unpublished data) [51]. An increase in the intracellular Ca^2+^ concentrations affects cell metabolism, proliferation, and apoptosis in cancer cells [52,53]. The four cell lines used in this study had major differences in their basal phenotype, which seemed to affect and dictate the response to digitoxin treatment. BxPC-3 and Panc-1, both derived from primary human tumors, were most affected by digitoxin treatment regarding their negative effects on cell viability. CFPAC-1, derived from a liver metastasis, and AsPC-1, from an ascites metastasis (mouse xenograft), were less affected by digitoxin. In contrast with the general negative effects of digitoxin on cancer cell viability, we detected differences in metabolism, ROS production, and proliferation in the PDAC cell lines [54,55].

The four investigated cell lines differed in their mutations in *KRAS* or *BRAF,* potentially affecting the downstream signaling pathways of PI3K/Akt and/or Raf/MEK/ERK [56,57,58,59]. CFPAC-1 with the *KRAS ^p.G12V^* and BxPC-3 with *BRAF* mutation had a relatively high rate of glycolysis under basal conditions and also a high transcriptional expression of *GLUT1, HK2*, and *PKM2*. These genes are often found to be highly expressed in primary pancreatic tumors with the *KRAS ^p.G12V^* mutation [46]. Fritsche-Guenther et al. concluded that colorectal carcinoma cells with the *KRAS ^p.G12V^* mutation are highly glycolytic [60]. According to Yun et al., colorectal cancer cells with *KRAS* and *BRAF* mutations have a constitutively activated *GLUT1* and enhanced glycolysis, in contrast with cells with mutations in *PIK3CA* (the catalytic part of PI3K) [44]. Panc-1 and AsPC-1 with the *KRAS ^p.G12D^* mutation, had a lower basal rate of glycolysis and a low transcriptional expression of the genes important for glycolysis and glucose transport.

With digitoxin treatment, the BxPC-3 and CFPAC-1 cells maintained the rate of glycolysis, while the glycolysis rate decreased in Panc-1 and AsPC-1. However, digitoxin treatment did not affect the phosphorylation of Akt (PI3K/Akt pathway) or ERK1/2 (Raf/MEK/ERK1/2 pathway) in the cell lines in this study. Therefore, we conclude that the changed glycolysis rate is controlled via other pathways.

The high glycolytic activity in BxPC-3 and CFPAC-1 could be the reason for the relatively low increase in intracellular Ca^2+^ in these two cell lines. Maintaining a low concentration of intracellular Ca^2+^ is fundamental for the normal function of the cell and is mainly managed by PMCA [61]. As shown in a study by James et al., 2020, PMCA function depends on ATP produced by glycolysis in PDAC [52].

In contrast, Panc-1 with its low glycolysis rate was not able to provide PMCA with enough ATP, hence the large increase of intracellular Ca^2+^. Mitochondria are also important in the regulation of calcium homeostasis [23,62,63]. When the intracellular concentrations of Ca^2+^ increase, some of the excess Ca^2+^ is transported into the mitochondria, causing upregulation of the citric acid cycle and oxidative phosphorylation [62]. If the intracellular Ca^2+^ increases too much, it will induce apoptosis [64]. 

Digitoxin treatment caused an increase in ROS production in all four cell lines, but to the highest degree in BxPC-3 and Panc-1. As oxidative phosphorylation is the major producer of ROS, we hypothesized that the increase in ROS was due to the increased activity of oxidative phosphorylation caused by Ca^2+^. ROS is important for controlling the redox state of the cell and, further, to control the cell cycle [26,28,31]. We found that BxPC-3 and Panc-1 have a high proportion of cells in S/G2 phase. Cells in this phase are more dependent on oxidative phosphorylation than cells in the other phases in the cell cycle [29]. The large proportion of cells in S/G2 in Panc-1 make this cell line highly dependent on oxidative phosphorylation for energy and molecules for nucleotide and lipid synthesis [65]. Panc-1 also had an increased proportion of cells in S/G2 with digitoxin treatment, which was reflected in the increase in oxidative phosphorylation (ROS) and decrease in the glycolysis rate. An increase in the proportion of cells in the S/G2-phase might imply DNA damage. In a study on HeLa cells by Gan et al., 2020, they found digitoxin to cause cell arrest in G2 due to DNA double strand breaks [66].

Both BxPC-3 and CFPAC-1 had an increase in cells in the G0/G1 phase when treated with digitoxin. These cell lines were similar in their basal rate and response in glycolysis rate to digitoxin treatment, and during treatment, they increased the proportion of cells in G0/G1. The increased proportions of cells in G0/G1 in BxPC-3 and CFPAC-1 with digitoxin treatment could be due to an arrest in G1. In earlier studies, cardiac glycosides have been shown to introduce arrest in G1 both in non-small cell lung cancer and in large cell lung cancer [67,68]. As the cells in the G1 phase are dependent on energy and products from glycolysis, this is a feasible explanation for the maintained rate of glycolysis in BxPC-3 and CFPAC-1 [69]. 

AsPC-1 with *KRAS ^p.G12D^* was outstanding because of the exceptionally low effects of digitoxin treatment compared with the other cell lines. AsPC-1 had a low basal rate for both glycolysis and oxidative phosphorylation, which could reflect a high proportion of cells in G0/G1. We believe that most of the cells in AsPC-1 were in the quiescent G0 phase, with only basal oxidative metabolism. They were slow growing and had a longer doubling time compared with the other cell lines, which supports the idea of a quiescent mode. Invading cancer cells are quite insensitive to digitoxin and have most of their cells in G0 [70]. The high chemoresistance found in invading cancer cells was probably due to the fact that they are mainly in the G0/G1 phase [27].

We found large differences in response to digitoxin in the cell lines in this study. BxPC-3 and CFPAC-1 seemed to be halted in G1, Panc-1 in G2 in the cell cycle, while AsPC-1 was unaffected. Wang et al., 2021, also found large differences in response to digoxin treatment between two cell lines derived from non-small cell lung cancer (NSCLC)—one of the cell lines was arrested in G1 and the other in G2 [68].

We therefore conclude that the beneficial effects of digitoxin on pancreatic cancer cells are the most pronounced in primary, highly proliferating and non-migrating tumors. In addition, when using cell lines as model systems in cancer research, it is important to be aware of the phenotypic/genotypic differences between the cell lines. 

## 4. Materials and Methods

### 4.1. Cell Culturing

Four pancreatic cancer cell lines were used: BxPC-3, CFPAC-1, Panc-1, and AsPC-1 (ATCC, Wesel, Germany) (Table 1).

BxPC-3 and AsPC-1 were grown in RPMI-1640 medium (Sigma-Aldrich, St. Louis, MO, USA) supplemented with 1% HEPES and 1% sodium pyruvate. CFPAC-1 was grown in Iscoves Modified Dulbecco’s Medium (IMDM) (Sigma-Aldrich, St. Louis, MO, USA), Panc-1 in Dulbecco´s Modified Eagle Medium (DMEM) (Sigma-Aldrich, St. Louis, MO, USA) with the addition of 1% L-glutamine. All of the media were additionally supplemented with 10% FBS and 1% PEST (Sigma-Aldrich, St. Louis, MO, USA). PCR was used for testing the cell lines for mycoplasma infection (LookOut Mycoplasma PCR Detection Kit #MP0035, Sigma-Aldrich, St. Louis, MO, USA).

### 4.2. Seeding and Treatment with Digitoxin

All of the analyses were performed in 96-well plates and after 48 h of treatment with digitoxin (Sigma-Aldrich, St. Louis, MO, USA), 5000 cells were seeded in 100 µL complete growth medium and incubated at 37 °C with 5% CO_2_ to a sub-confluent monolayer. After 20 h of incubation, the media were removed and 100 µL new media containing digitoxin were added to the cells. The control cells only received new media. The concentrations of digitoxin used were 0 nM (controls), 1 nM, 10 nM, 25 nM, 40 nM, and 100 nM (human therapeutic range, 25–40 nM). Cells were further incubated at 37 °C with 5% CO_2_ for 48 h. 

### 4.3. RNA-Extraction, cDNA Synthesis, and Real-Time PCR

For each RNA extraction, 1 × 10^5^ cells were seeded in 6-well plates in 2.4 mL complete medium, and they were incubated for 20 h at 37 °C with 5% CO_2_ to a sub-confluent monolayer. After 20 h, the media were removed and new media were added together with digitoxin in the same concentrations as previously mentioned. The control cells only received new media.

The cells were treated for 48 h with digitoxin, and RNA was extracted using the RNeasy Mini Kit (Qiagen, Hilden, Germany) according to the manufacturer’s protocol. For cDNA synthesis, 1 µg of RNA from each sample was used in High-Capacity cDNA Reverse Transcription Kit Reagent (Thermo Fisher Scientific, Waltham, MA, USA).

cDNA corresponding to 5 ng of RNA was used in each qPCR reaction. qPCR was performed on a Pikoreal qPCR System (Thermo Fischer Scientific Waltham, MA, USA) in duplicate for the TaqMan target transcripts (TaqMan Gene Expression Assays, Applied Biosystems, Foster City, CA, USA), using TaqMan™ Gene Expression Master Mix (4369016, Applied Biosystems, Foster City, CA, USA) for the genes *GLUT1* (Assay ID: Hs00892681), *HK2* (Assay ID: Hs00606086), *LDHA* (Assay ID: Hs03405707), and the reference gene *PMM1* (Assay ID: Hs00963626_m1).

A 5 µL reaction mixture was prepared with 1.3 µM of each primer, 5 ng of cDNA, and 2x Fast SYBR™ Green Master Mix (4385617, Applied Biosystems, Foster City, CA, USA). Primers for the gene *PKM2* were forward 5′-GTCTGGAGAAACAGCCAAGG and reverse 5′-CGGAGTTCCTCGAATAGTG. The PCR cycling conditions were 95 °C for 7 min, 40 cycles of 95 °C for 10 s, and 60 °C for 15 s, followed by a melt curve setting. Quantitative gene expression data were normalized to the expression level of the human reference gene *PMM1* (Figure S1: Gene expression).

### 4.4. Protein Extraction and Western Blot (WB)

Cells were seeded in a quantity of 5 × 10^5^ cells per 75 cm^2^ flask and incubated for 20 h at 37 °C with 5% CO_2_; thereafter, the old media were removed and new media with digitoxin in different concentrations were added. The controls only received new, complete media. Proteins were extracted after 48 h of treatment with digitoxin and the cells were lysed in a lysis buffer (FNN0011, Thermo Fisher Scientific, Waltham, MA, USA) supplemented with phenylmethylsulphonyl fluoride (PMSF) (36978, Thermo Fisher Scientific, Waltham, MA, USA) and protein inhibitor cocktail (P2714, Sigma-Aldrich, St. Louis, MO, USA). The protein concentration in the samples was determined using the Pierce™ BCA Protein Assay Kit (Thermo Fisher Scientific, Waltham, MA, USA).

The samples were separated using SDS-PAGE (sodium dodecyl sulfate-polyacrylamide electrophoresis) (8–16% gel), blotted to PVDF membranes, blocked in PBST with 5% milk, and incubated for 16–20 h with primary antibodies (Akt #9272, pAkt #9271, ERK1/2 #4695, and pERK1/2 #4695) (Cell signaling Technology, Danvers, MA, USA) (1:1000) at 4 °C. The membranes were further incubated with a secondary antibody in PBST with 5% milk, Alexa Fluor 488 (1:2500). The protein expression was measured using the ChemiDoc System (BioRad Laboratories Inc., Hercules, CA, USA). The protein expression was normalized to the total protein for each sample (Figure S2: WB).

### 4.5. Cell Viability Assay

Cell viability analysis was performed with the CellTiter 96^®^ AQ_ueous_ One Solution Cell Proliferation Assay (MTS) (Promega, Madison, WI, USA). This is a colorimetric method for determination of the number of viable cells. After 48 h of incubation with digitoxin, 20 µL of MTS tetrazolium was added to each well and they were then incubated for 1 h. The quantity of the formazan product was measured using a spectrophotometer at 490 nm (FLUOstar Omega, BMG Labtech, Ortenberg, Germany). The value was directly proportional to the number of living cells in the cell culture. All of the assays were performed in octuplicates and were repeated five times (Table S1: MTS).

### 4.6. Caspase 3/7 Assay

Cells were seeded in black-walled 96-well cell culture plates and were treated with digitoxin, as previously described, and incubated for 48 h. The endpoint assay in the CellEvent™ Caspase-3/7 Green Detection Reagent kit (C10423, Invitrogen/ThermoFisher Scientific, Waltham, MA, USA) was performed. Reagents in this assay consisted of a DEVD peptide conjugated to a fluorescent dye binding to nucleic acids. The activation of Caspase 3 and/or Caspase 7 cleaved the conjugated DEVD peptide and the nucleic acid binding dye became fluorescent when binding to the DNA. The fluorescence intensity was measured with a microplate reader, FluoStar, at Ex/Em 485/520 (FLUOstar Omega, BMG Labtech, Ortenberg, Germany). The assay was performed in triplicate and repeated three times.

### 4.7. Extracellular Lactate-Aerobic Glycolysis

Cells were seeded in 96-well plates and after 48 h of treatment with digitoxin, 5 µL of media was diluted 1:20 in PBS and kept in –20 °C until the analysis. The samples were further diluted to a final dilution of 1:800 and were analyzed in by Lactate-Glo™ Assay (Promega, Madison, WI, USA). Luminescence was measured in a microplate reader, FluoStar. The assay was performed in duplicate and was repeated twice.

### 4.8. Intracellular ROS

Cells were seeded and treated with digitoxin in triplicate for 48 h, as described earlier. The oxidative stress was measured using the CellROX green reagent (C10444, Invitrogen/ThermoFisher Scientific, Waltham, MA, USA). The dye has a bright green fluorescence when oxidated by ROS and after subsequent binding to DNA. The fluorescence intensity was measured with a microplate reader, Fluostar, at Ex/Em = 485/520 (FLUOstar Omega, BMG Labtech, Ortenberg, Germany). The assay was performed for two biological replicates and two technical replicates.

### 4.9. Cell Cycle Analysis

The Cell-Clock assay (Biocolor, Carrickfergus, UK) was used to assess the basal proliferation status and the effects of digitoxin on the cell cycle in the four cell lines. This is a system to measure the four phases, G0-G1, S, G2, and M, of the cell cycle in live cells. Cells were seeded in 96-well plates, treated with digitoxin for 48 h, and incubated with the provided redox dye for 1 h at 37 °C; then, they were manually evaluated by two researchers blinded to the actual treatment. The ratio of each phase was calculated (Table S2: Cell cycle).

### 4.10. Statistical Analysis

Statistical analysis was performed using IBM SPSS Statistics 27.0 (Armonk, NY, USA: IBM Corp), Student´s t-test (paired, two-tailed) for cell viability, caspase 3/7, and Cell-Clock results to confirm significance among treatments, compared with the control. The effect of digitoxin treatment on the transcriptional expression, protein expression, and lactate production was assessed by linear regression. Significance values were * *p* ≤ 0.05, ** *p* ≤ 0.01, and *** *p* ≤ 0.001.

## 5. Conclusions

The high malignancy and poor prognosis of PDAC, together with increasing patient numbers, call for the development of new treatment strategies. Increased knowledge about the effect that digitoxin exerts on PDAC cells is needed to validate its use for the treatment of pancreatic cancer. In order to obtain a maximal effect on cancer cell viability, a combination of treatments affecting metabolism and proliferation in cancer is preferable. 

We have shown that digitoxin can act as an anti-cancer agent in several pancreatic cancer cell lines, but it affects metabolism, ROS production, and proliferation differently depending on the cell type. Cell metabolism and proliferation are closely connected because of the changing demands in the different phases in the cell cycle, and the proportion of cells in the S-phase seems to determine the success of digitoxin treatment. 

Our results show that BxPC-3 and Panc-1, derived from primary tumors, have a high proportion of cells in the building/synthesizing phases in the cell cycle, and are also the most vulnerable to digitoxin treatment. Knowing the metabolic and proliferative pattern in human tumors will be critical for choosing the correct treatment. 

## Figures and Tables

**Table 1:** Pancreatic cancer cell lines.

ATCC^®^ No.	Name	Histology	Tumour Source	Mutant Gene
CRL-1687™	BxPC-3	Adenocarcinoma	Primary	*BRAF, CDKN2A, MAP2K4, SMAD4, TP53*
CRL-1918™	CFPAC-1	Ductal adenocarcinoma	Metastasis, liver	*KRAS ^p. G12V^*, *SMAD4, TP53*
CRL-1469™	Panc-1	Adenocarcinoma	Primary	*KRAS ^p. G12D^, TP53*
CRL-1682™	AsPC-1	Adenocarcinoma	Xenograft	*KRAS ^p. G12D^, CDKN2A, MAP2K4, FBXW7, TP53*

Pancreatic cancer cell lines, classification by ATCC (ATCC – LGC Standards GmbH, Wesel, Germany).

## Data Availability

Data are contained within the article or in the Supplementary Material.

## Supplementary Materials

The following supporting information can be downloaded at: https://www.mdpi.com/article/10.3390/ijms23158237/s1.

## References

[1] Becker A.E., Hernandez Y.G., Frucht H., Lucas A.L. (2014). Pancreatic ductal adenocarcinoma: Risk factors, screening, and early detection. World J. Gastroenterol..

[2] Ansari D., Gustafsson A., Andersson R. (2015). Update on the management of pancreatic cancer: Surgery is not enough. World J. Gastroenterol..

[3] Pereira N.P., Corrêa J.R. (2018). Pancreatic cancer: Treatment approaches and trends. J. cancer metastatis treat..

[4] Fouad Y.A., Aanei C. (2017). Revisiting the hallmarks of cancer. Am. J. Cancer Res..

[5] Hanahan D., Weinberg R.A. (2011). Hallmarks of Cancer: The Next Generation. Cell.

[6] Duronio R.J., Xiong Y. (2013). Signaling pathways that control cell proliferation. Cold Spring Harb. Perspect. Biol..

[7] Diaz-Moralli S., Tarrado-Castellarnau M., Miranda A., Cascante M. (2013). Targeting cell cycle regulation in cancer therapy. Pharmacol. Ther..

[8] Kumari S., Badana A.K., G M.M., G S., Malla R. (2018). Reactive Oxygen Species: A Key Constituent in Cancer Survival. Biomark. Insights.

[9] Yang X.S., Xu Z.W., Yi T.L., Xu R.C., Li J., Zhang W.B., Zhang S., Sun H.T., Yu Z.Q., Xu H.X. (2018). Ouabain suppresses the growth and migration abilities of glioma U-87MG cells through inhibiting the Akt/mTOR signaling pathway and downregulating the expression of HIF-1α. Mol. Med. Rep..

[10] Zhang H., Qian D.Z., Tan Y.S., Lee K., Gao P., Ren Y.R., Rey S., Hammers H., Chang D., Pili R. (2008). Digoxin and other cardiac glycosides inhibit HIF-1alpha synthesis and block tumor growth. Proc. Natl. Acad. Sci. USA.

[11] Haux J. (1999). Digitoxin is a potential anticancer agent for several types of cancer. Med. Hypotheses..

[12] Haux J., Klepp O., Spigset O., Tretli S. (2001). Digitoxin medication and cancer; case control and internal dose-response studies. BMC Cancer.

[13] Shapiro W., Taubert K., Narahara K. (1972). Nonradioactive Serum Digoxin and Digitoxin Levels. Arch. Intern. Med..

[14] Kepp O., Menger L., Vacchelli E., Adjemian S., Martins I., Ma Y., Sukkurwala A.Q., Michaud M., Galluzzi L., Zitvogel L. (2012). Anticancer activity of cardiac glycosides: At the frontier between cell-autonomous and immunological effects. Oncoimmunology.

[15] Frankel A.E., Eskiocak U., Gill J.G., Yuan S., Ramesh V., Froehlich T.W., Ahn C., Morrison S.J. (2017). Digoxin Plus Trametinib Therapy Achieves Disease Control in BRAF Wild-Type Metastatic Melanoma Patients. Neoplasia.

[16] Li Q., Pogwizd S.M., Prabhu S.D., Zhou L. (2014). Inhibiting Na^+^/K^+^ ATPase can impair mitochondrial energetics and induce abnormal Ca^2+^ cycling and automaticity in guinea pig cardiomyocytes. PLoS ONE.

[17] Kembro J.M., Aon M.A., Winslow R.L., O′Rourke B., Cortassa S. (2013). Integrating mitochondrial energetics, redox and ROS metabolic networks: A two-compartment model. Biophys. J..

[18] Bae Y.S., Oh H., Rhee S.G., Yoo Y.D. (2011). Regulation of reactive oxygen species generation in cell signaling. Mol. Cells.

[19] Tu B.P., Kudlicki A., Rowicka M., McKnight S.L. (2005). Logic of the yeast metabolic cycle: Temporal compartmentalization of cellular processes. Science.

[20] Kaludercic N., Deshwal S., Lisa F.D. (2014). Reactive oxygen species and redox compartmentalization. Front. Physiol..

[21] Menon S.G., Goswami P.C. (2007). A redox cycle within the cell cycle: Ring in the old with the new. Oncogene.

[22] Pizzino G., Irrera N., Cucinotta M., Pallio G., Mannino F., Arcoraci V., Squadrito F., Altavilla D., Bitto A. (2017). Oxidative Stress: Harms and Benefits for Human Health. Oxid. Med. Cell. Longev..

[23] Liu T., O′Rourke B. (2009). Regulation of mitochondrial Ca^2+^ and its effects on energetics and redox balance in normal and failing heart. J. Bioenerg. Biomembr..

[24] Cortassa S., Aon M.A., Marbán E., Winslow R.L., O′Rourke B. (2003). An integrated model of cardiac mitochondrial energy metabolism and calcium dynamics. Biophys. J..

[25] Lo Y.-C., Senese S., France B., Gholkar A.A., Damoiseaux R., Torres J.Z. (2017). Computational Cell Cycle Profiling of Cancer Cells for Prioritizing FDA-Approved Drugs with Repurposing Potential. Sci. Rep..

[26] Burhans W.C., Heintz N.H. (2009). The cell cycle is a redox cycle: Linking phase-specific targets to cell fate. Free Radic. Biol. Med..

[27] Yano S., Miwa S., Mii S., Hiroshima Y., Uehara F., Yamamoto M., Kishimoto H., Tazawa H., Bouvet M., Fujiwara T. (2014). Invading cancer cells are predominantly in G0/G1 resulting in chemoresistance demonstrated by real-time FUCCI imaging. Cell Cycle.

[28] da Veiga Moreira J., Peres S., Steyaert J.-M., Bigan E., Paulevé L., Nogueira M.L., Schwartz L. (2015). Cell cycle progression is regulated by intertwined redox oscillators. Theor. Biol. Med. Model..

[29] Bao Y., Mukai K., Hishiki T., Kubo A., Ohmura M., Sugiura Y., Matsuura T., Nagahata Y., Hayakawa N., Yamamoto T. (2013). Energy management by enhanced glycolysis in G1-phase in human colon cancer cells in vitro and in vivo. Mol. Cancer Res..

[30] Beresford M.J., Wilson G.D., Makris A. (2006). Measuring proliferation in breast cancer: Practicalities and applications. Breast Cancer Res..

[31] Chiu J., Dawes I.W. (2012). Redox control of cell proliferation. Trends Cell Biol..

[32] Gable M.E., Abdallah S.L., Najjar S.M., Liu L., Askari A. (2014). Digitalis-induced cell signaling by the sodium pump: On the relation of Src to Na(+)/K(+)-ATPase. Biochem. Biophys. Res. Commun..

[33] Tian J., Cai T., Yuan Z., Wang H., Liu L., Haas M., Maksimova E., Huang X.Y., Xie Z.J. (2006). Binding of Src to Na^+^/K_+_-ATPase forms a functional signaling complex. Mol. Biol. Cell.

[34] Liang M., Tian J., Liu L., Pierre S., Liu J., Shapiro J., Xie Z.-J. (2007). Identification of a Pool of Non-pumping Na/K-ATPase. J. Biol. Chem..

[35] Souza e Souza K.F.C., Moraes B.P.T., Paixao I.C.N.D.P., Burth P., Silva A.R., Gonçalves-de-Albuquerque C.F. (2021). Na^+^/K^+^-ATPase as a Target of Cardiac Glycosides for the Treatment of SARS-CoV-2 Infection. Front. Pharmacol..

[36] Witkiewicz A.K., McMillan E.A., Balaji U., Baek G., Lin W.-C., Mansour J., Mollaee M., Wagner K.-U., Koduru P., Yopp A. (2015). Whole-exome sequencing of pancreatic cancer defines genetic diversity and therapeutic targets. Nat. Commun..

[37] Connor A.A., Denroche R.E., Jang G.H., Lemire M., Zhang A., Chan-Seng-Yue M., Wilson G., Grant R.C., Merico D., Lungu I. (2019). Integration of Genomic and Transcriptional Features in Pancreatic Cancer Reveals Increased Cell Cycle Progression in Metastases. Cancer Cell.

[38] Gu Y., Yang D., Zou J., Ma W., Wu R., Zhao W., Zhang Y., Xiao H., Gong X., Zhang M. (2010). Systematic Interpretation of Comutated Genes in Large-Scale Cancer Mutation Profiles. Mol. Cancer Ther..

[39] Waters A.M., Der C.J. (2018). KRAS: The Critical Driver and Therapeutic Target for Pancreatic Cancer. Cold Spring Harb. Perspect. Med..

[40] Bryant K.L., Mancias J.D., Kimmelman A.C., Der C.J. (2014). KRAS: Feeding pancreatic cancer proliferation. Trends Biochem. Sci..

[41] Polosukhina D., Love H.D., Correa H., Su Z., Dahlman K.B., Pao W., Moses H.L., Arteaga C.L., Lovvorn H.N., Zent R. (2017). Functional KRAS mutations and a potential role for PI3K/AKT activation in Wilms tumors. Mol. Oncol..

[42] Liu P., Wang Y., Li X. (2019). Targeting the untargetable KRAS in cancer therapy. Acta. Pharm. Sin. B.

[43] Dayton T.L., Jacks T., Vander Heiden M.G. (2016). PKM2, cancer metabolism, and the road ahead. EMBO Rep..

[44] Yun J., Rago C., Cheong I., Pagliarini R., Angenendt P., Rajagopalan H., Schmidt K., Willson J.K., Markowitz S., Zhou S. (2009). Glucose deprivation contributes to the development of KRAS pathway mutations in tumor cells. Science.

[45] Graziano F., Ruzzo A., Giacomini E., Ricciardi T., Aprile G., Loupakis F., Lorenzini P., Ongaro E., Zoratto F., Catalano V. (2017). Glycolysis gene expression analysis and selective metabolic advantage in the clinical progression of colorectal cancer. Pharmacogenomics J..

[46] Anderson M., Marayati R., Moffitt R., Yeh J.J. (2017). Hexokinase 2 promotes tumor growth and metastasis by regulating lactate production in pancreatic cancer. Oncotarget.

[47] Xie H., Hanai J., Ren J.G., Kats L., Burgess K., Bhargava P., Signoretti S., Billiard J., Duffy K.J., Grant A. (2014). Targeting lactate dehydrogenase—A inhibits tumorigenesis and tumor progression in mouse models of lung cancer and impacts tumor-initiating cells. Cell Metab..

[48] Koukourakis M.I., Giatromanolaki A., Simopoulos C., Polychronidis A., Sivridis E. (2005). Lactate dehydrogenase 5 (LDH5) relates to up-regulated hypoxia inducible factor pathway and metastasis in colorectal cancer. Clin. Exp. Metastasis.

[49] Mi C., Cao X., Ma K., Wei M., Xu W., Lin Y., Zhang J., Wang T.-Y. (2022). Digitoxin promotes apoptosis and inhibits proliferation and migration by reducing HIF-1α and STAT3 in KRAS mutant human colon cancer cells. Chem. Biol. Interact..

[50] Lee D.H., Cheul O.S., Giles A.J., Jung J., Gilbert M.R., Park D.M. (2017). Cardiac glycosides suppress the maintenance of stemness and malignancy via inhibiting HIF-1α in human glioma stem cells. Oncotarget.

[51] Lindholm H., Ejeskär K., Szekeres F. (2022). The Na^+^/K^+^-ATPase subunit α3 expression correlates to digitoxin treatment efficiency in pancreatic cancer cells. Med. Int..

[52] James A.D., Richardson D.A., Oh I.W., Sritangos P., Attard T., Barrett L., Bruce J.I.E. (2020). Cutting off the fuel supply to calcium pumps in pancreatic cancer cells: Role of pyruvate kinase-M2 (PKM2). Br. J. Cancer.

[53] Dejos C., Gkika D., Cantelmo A.R. (2020). The Two-Way Relationship Between Calcium and Metabolism in Cancer. Front. Cell Dev. Biol..

[54] Collisson E.A., Sadanandam A., Olson P., Gibb W.J., Truitt M., Gu S., Cooc J., Weinkle J., Kim G.E., Jakkula L. (2011). Subtypes of pancreatic ductal adenocarcinoma and their differing responses to therapy. Nat. Med..

[55] Daemen A., Peterson D., Sahu N., McCord R., Du X., Liu B., Kowanetz K., Hong R., Moffat J., Gao M. (2015). Metabolite profiling stratifies pancreatic ductal adenocarcinomas into subtypes with distinct sensitivities to metabolic inhibitors. Proc. Natl. Acad. Sci. USA.

[56] Riquelme E., Behrens C., Lin H.Y., Simon G., Papadimitrakopoulou V., Izzo J., Moran C., Kalhor N., Lee J.J., Minna J.D. (2016). Modulation of EZH2 Expression by MEK-ERK or PI3K-AKT Signaling in Lung Cancer Is Dictated by Different KRAS Oncogene Mutations. Cancer Res..

[57] Céspedes M.V., Sancho F.J., Guerrero S., Parreño M., Casanova I., Pavón M.A., Marcuello E., Trias M., Cascante M., Capellà G. (2006). K-ras Asp12 mutant neither interacts with Raf, nor signals through Erk and is less tumorigenic than K-ras Val12. Carcinogenesis.

[58] Hobbs G.A., Der C.J., Rossman K.L. (2016). RAS isoforms and mutations in cancer at a glance. J. Cell Sci..

[59] Ying H., Kimmelman A.C., Lyssiotis C.A., Hua S., Chu G.C., Fletcher-Sananikone E., Locasale J.W., Son J., Zhang H., Coloff J.L. (2012). Oncogenic Kras maintains pancreatic tumors through regulation of anabolic glucose metabolism. Cell.

[60] Fritsche-Guenther R., Zasada C., Mastrobuoni G., Royla N., Rainer R., Roßner F., Pietzke M., Klipp E., Sers C., Kempa S. (2018). Alterations of mTOR signaling impact metabolic stress resistance in colorectal carcinomas with BRAF and KRAS mutations. Sci. Rep..

[61] James A.D., Patel W., Butt Z., Adiamah M., Dakhel R., Latif A., Uggenti C., Swanton E., Imamura H., Siriwardena A.K. (2015). The Plasma Membrane Calcium Pump in Pancreatic Cancer Cells Exhibiting the Warburg Effect Relies on Glycolytic ATP. J. Biol. Chem..

[62] Rossi A., Pizzo P., Filadi R. (2019). Calcium, mitochondria and cell metabolism: A functional triangle in bioenergetics. Biochim. Biophys. Acta Mol. Cell. Res..

[63] Romero-Garcia S., Prado-Garcia H. (2019). Mitochondrial calcium: Transport and modulation of cellular processes in homeostasis and cancer (Review). Int. J. Oncol..

[64] Rizzuto R., Pinton P., Ferrari D., Chami M., Szabadkai G., Magalhães P.J., Virgilio F.D., Pozzan T. (2003). Calcium and apoptosis: Facts and hypotheses. Oncogene.

[65] Icard P., Fournel L., Wu Z., Alifano M., Lincet H. (2019). Interconnection between Metabolism and Cell Cycle in Cancer. Trends Biochem. Sci..

[66] Gan H., Qi M., Chan C., Leung P., Ye G., Lei Y., Liu A., Xue F., Liu D., Ye W. (2020). Digitoxin inhibits HeLa cell growth through the induction of G2/M cell cycle arrest and apoptosis in vitro and in vivo. Int. J. Oncol..

[67] Kaushik V., Yakisich J.S., Azad N., Kulkarni Y., Venkatadri R., Wright C., Rojanasakul Y., Iyer A.K.V. (2017). Anti-Tumor Effects of Cardiac Glycosides on Human Lung Cancer Cells and Lung Tumorspheres. J. Cell. Physiol..

[68] Wang Y., Hou Y., Hou L., Wang W., Li K., Zhang Z., Du B., Kong D. (2021). Digoxin exerts anticancer activity on human nonsmall cell lung cancer cells by blocking PI3K/Akt pathway. Biosci. Rep..

[69] Hoffman A., Spetner L.M., Burke M. (2008). Ramifications of a redox switch within a normal cell: Its absence in a cancer cell. Free Radic. Biol. Med..

[70] Park S.-Y., Nam J.-S. (2020). The force awakens: Metastatic dormant cancer cells. Exp. Mol. Med..

