# Supplementary figures and images for: Digitoxin Affects Metabolism, ROS Production and Proliferation in Pancreatic Cancer Cells Differently Depending on the Cell Phenotype
Source: Int J Mol Sci. 2022 Jul 26;23(15):8237. doi: 10.3390/ijms23158237 (PMC9331846; doi:10.3390/ijms23158237)

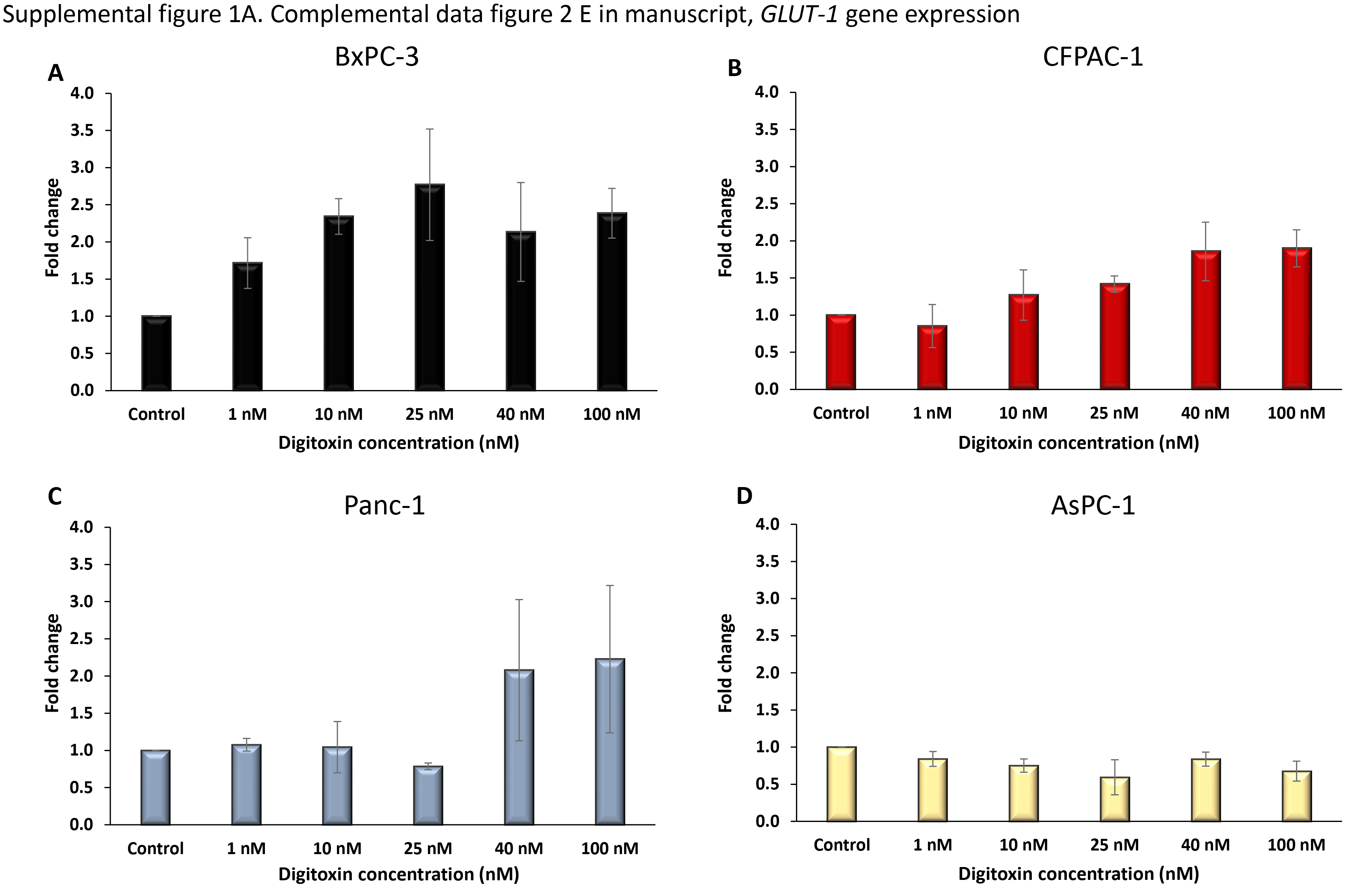

Supplement: Supplementary file 1 [file ijms-23-08237-s001.zip › Figure S1A.tif]

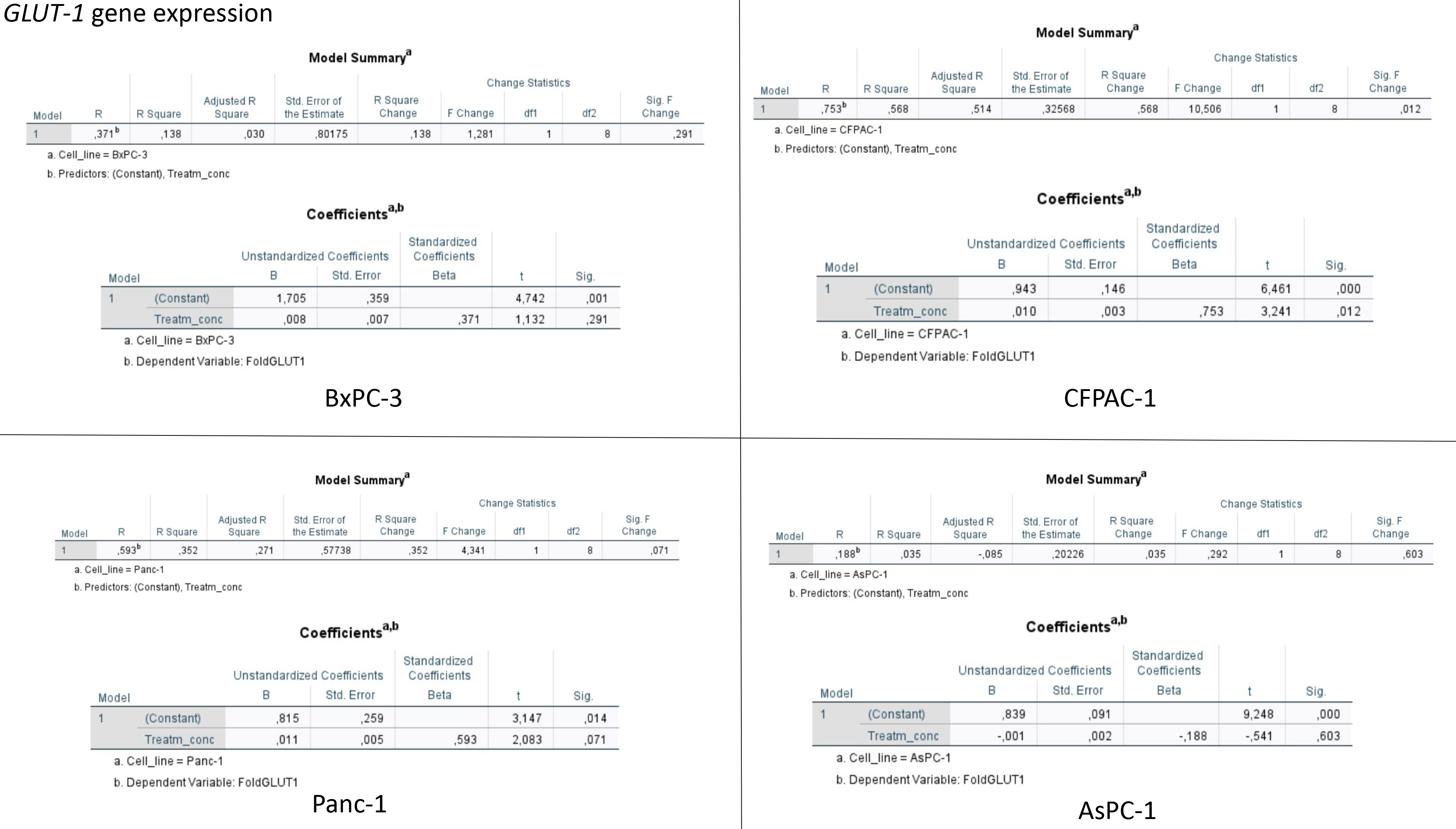

Supplement: Supplementary file 1 [file ijms-23-08237-s001.zip › Figure S1A_regr.tif]

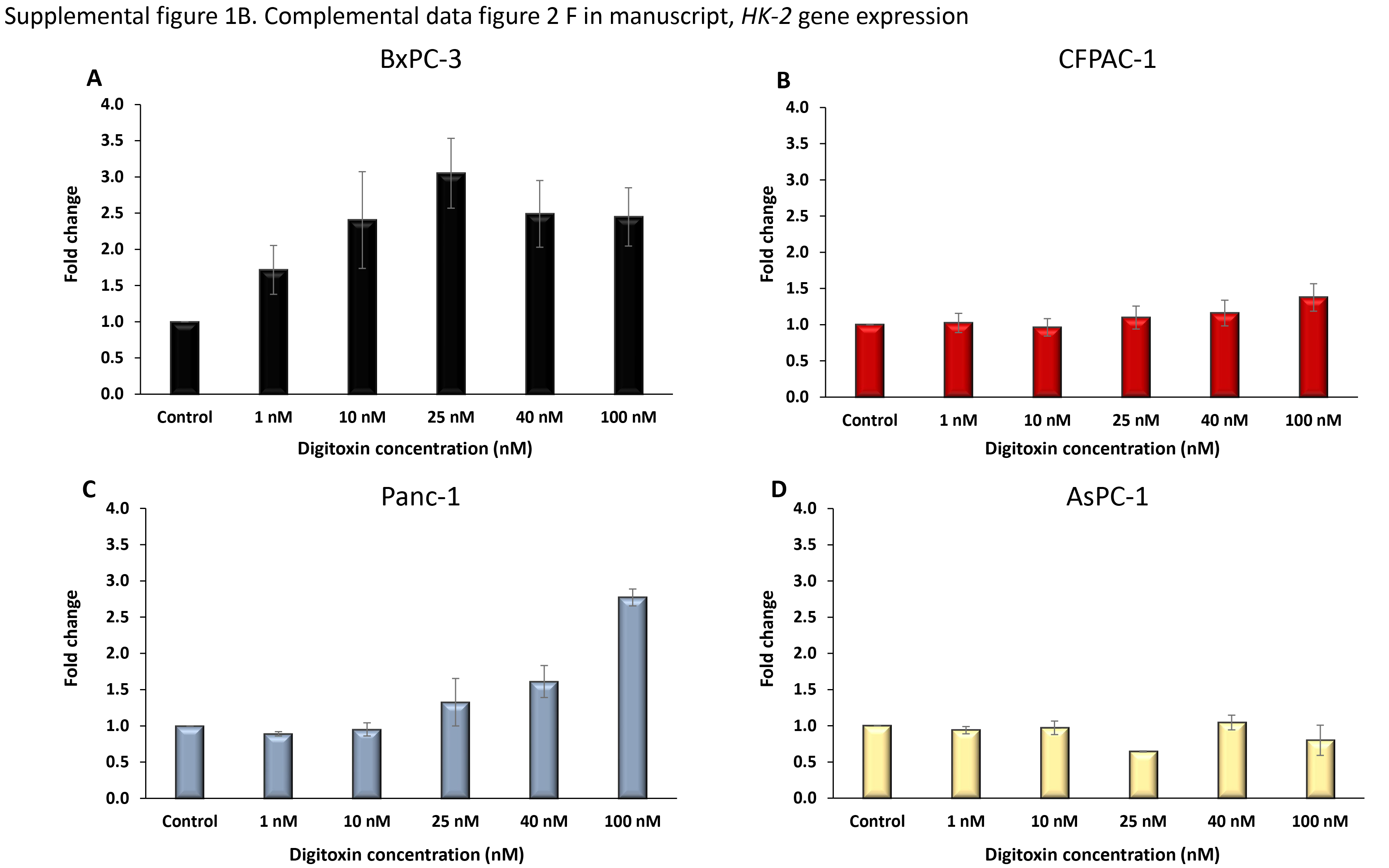

Supplement: Supplementary file 1 [file ijms-23-08237-s001.zip › Figure S1B.tif]

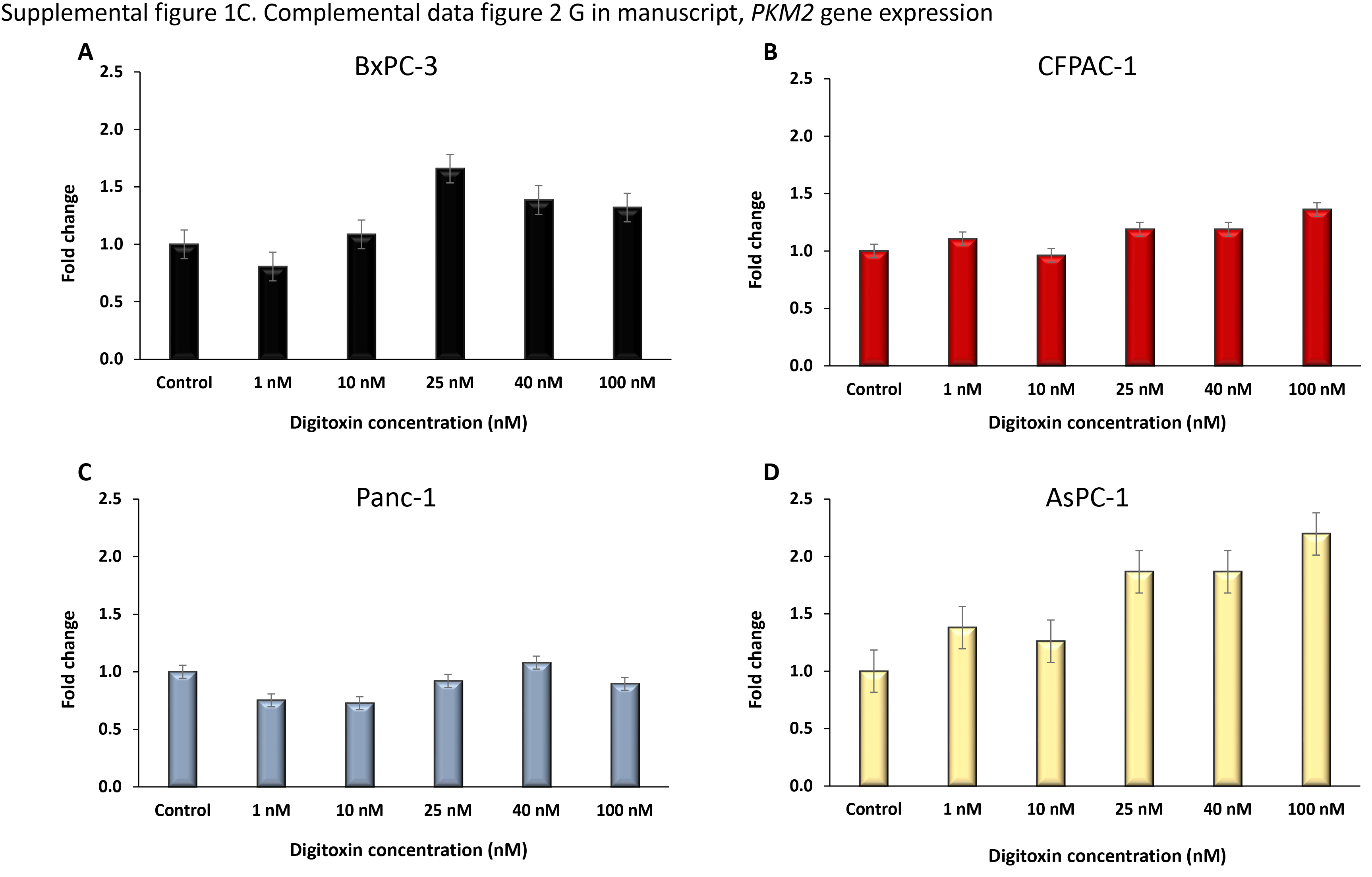

Supplement: Supplementary file 1 [file ijms-23-08237-s001.zip › Figure S1C.tif]

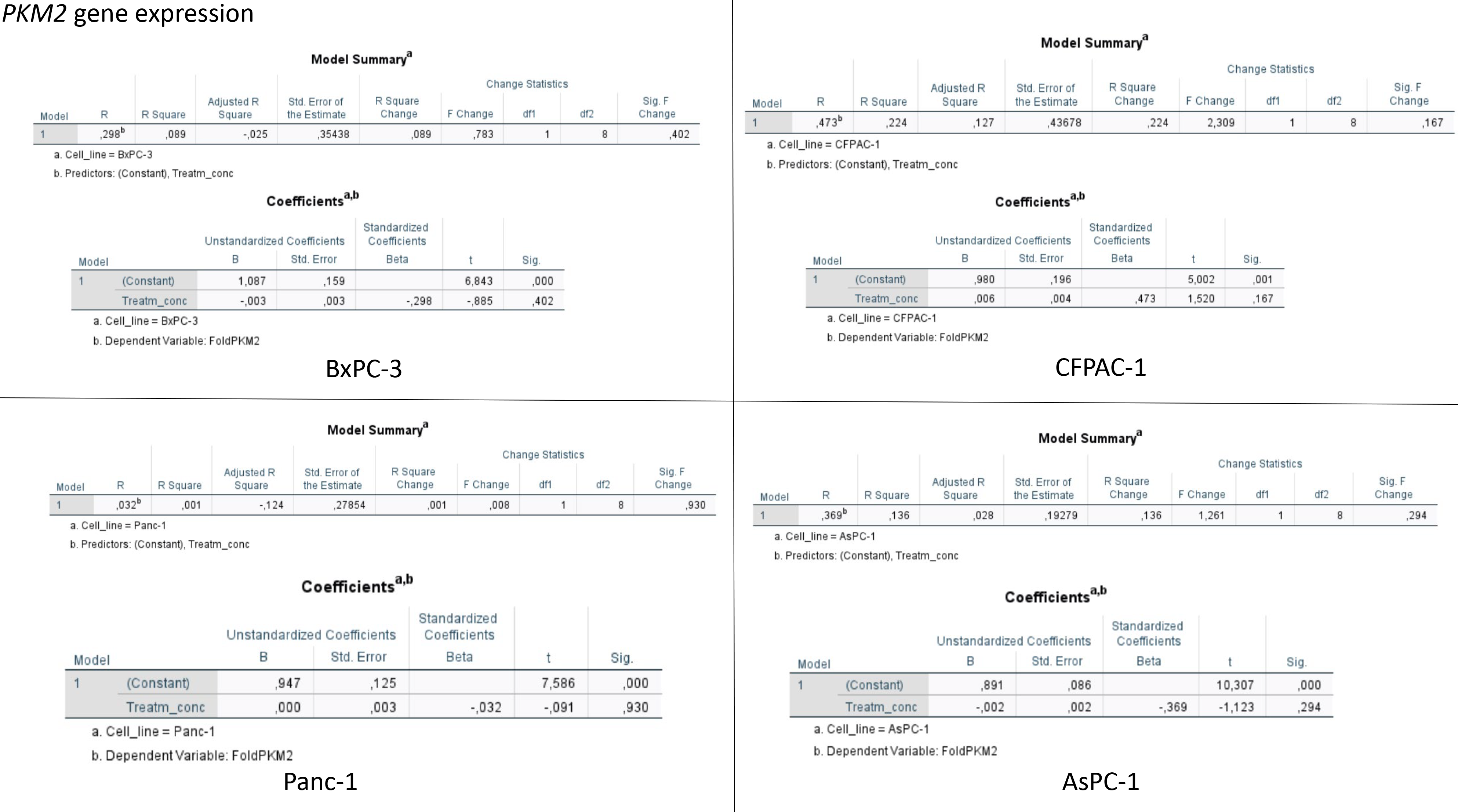

Supplement: Supplementary file 1 [file ijms-23-08237-s001.zip › Figure S1C_regr.tif]

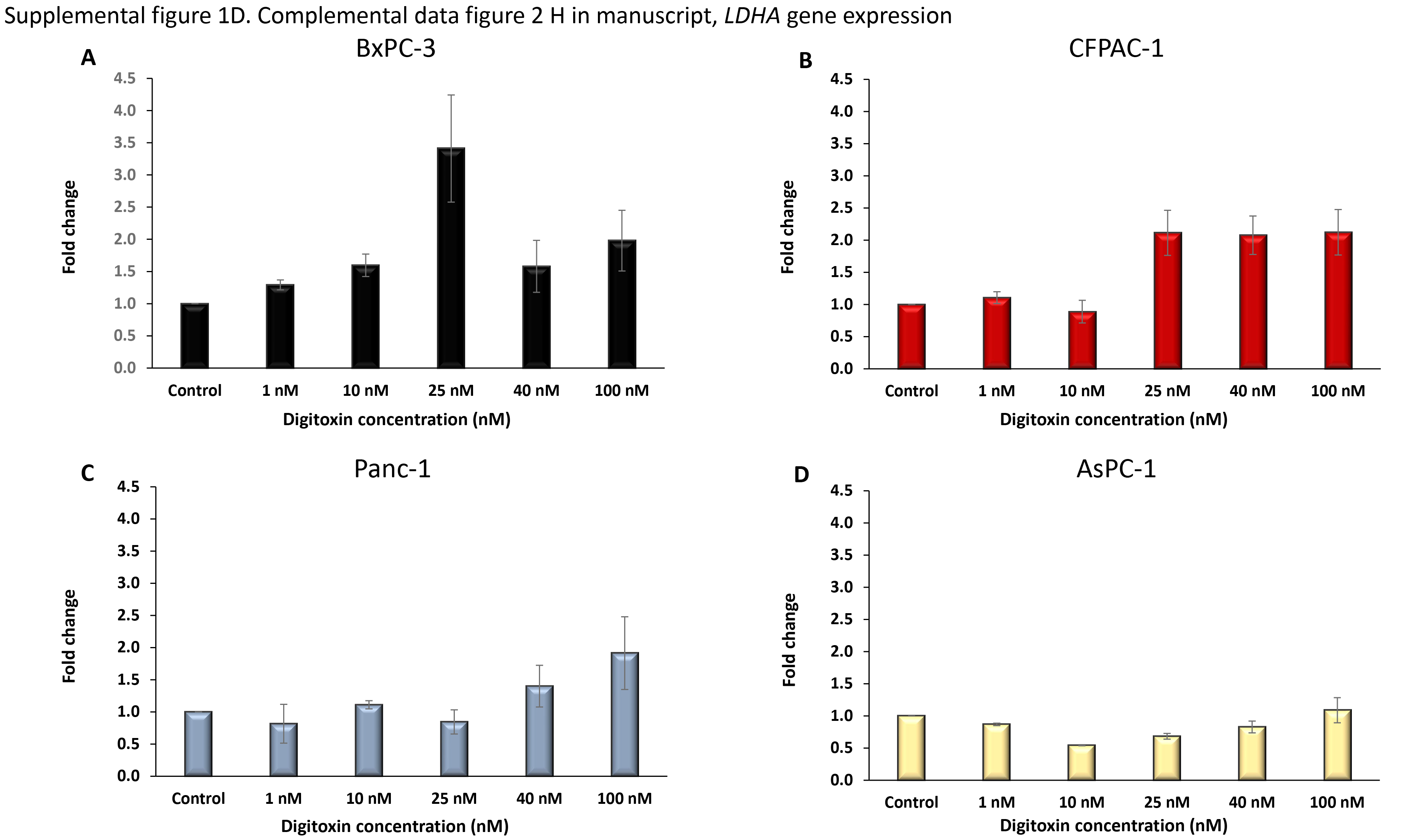

Supplement: Supplementary file 1 [file ijms-23-08237-s001.zip › Figure S1D.tif]

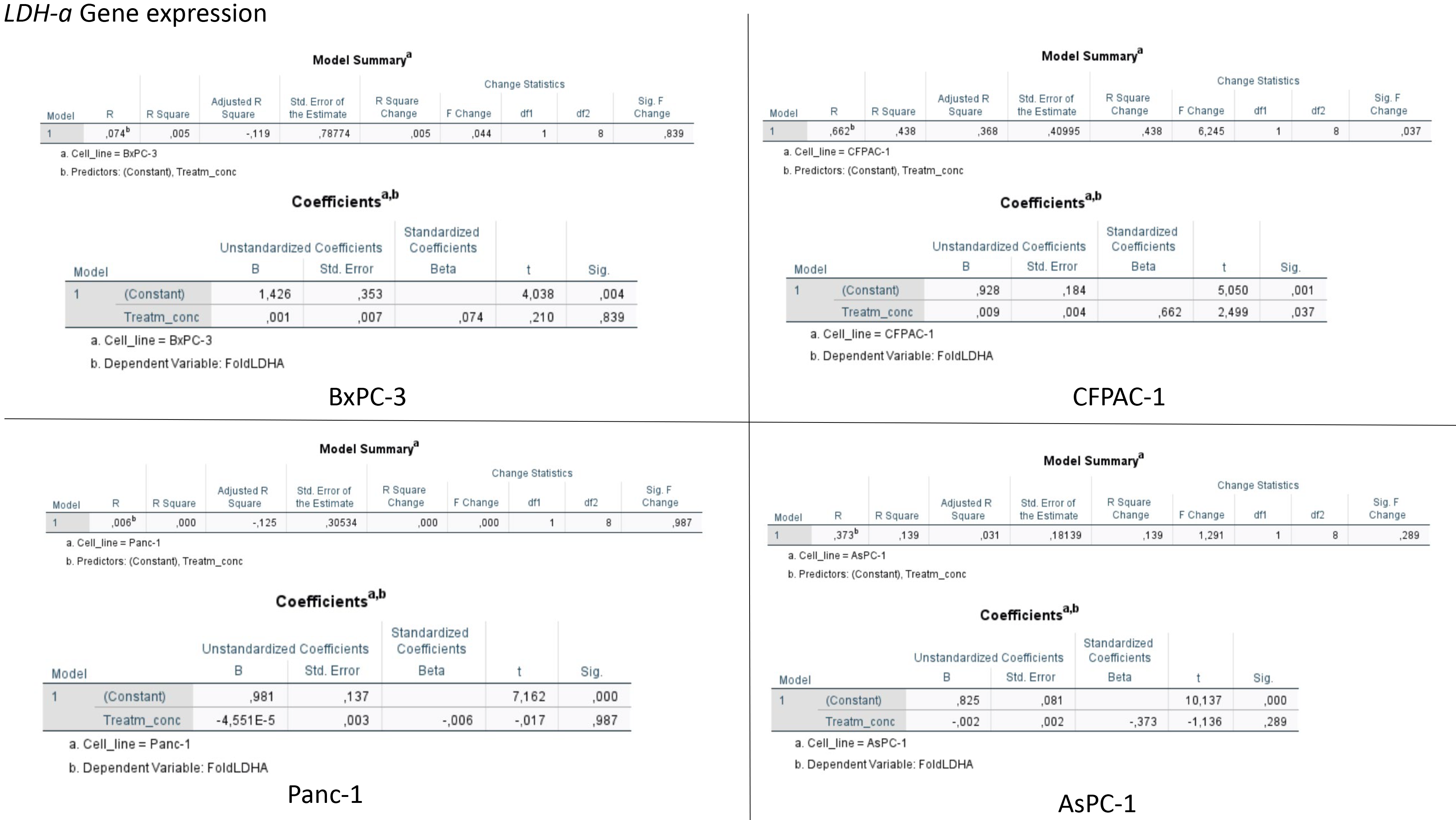

Supplement: Supplementary file 1 [file ijms-23-08237-s001.zip › Figure S1D_regr.tif]

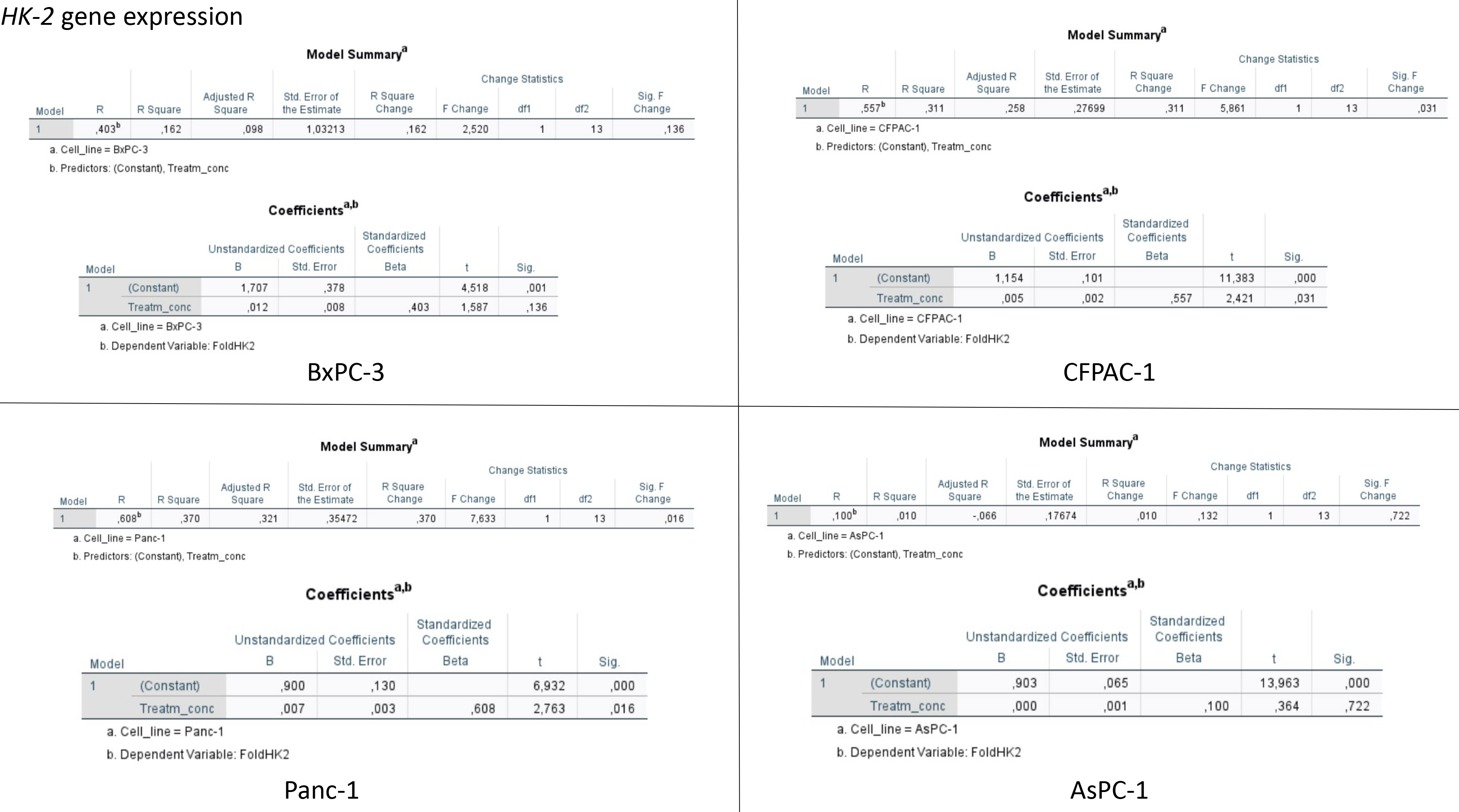

Supplement: Supplementary file 1 [file ijms-23-08237-s001.zip › Figure SB_regr.tif]
